# Inadvertent nucleotide sequence alterations during mutagenesis: highlighting the vulnerabilities in mouse transgenic technology

**DOI:** 10.1186/s43141-021-00130-5

**Published:** 2021-02-11

**Authors:** Anuran Ghosh, Rituparna Chakrabarti, Praphulla Chandra Shukla

**Affiliations:** School of Medical Science and Technology, Indian Institute of Technology Kharagpur, Kharagpur, West Bengal 721302 India

**Keywords:** Transgenic, Non-coding, Knockout, Fidelity

## Abstract

In the last three decades, researchers have utilized genome engineering to alter the DNA sequence in the living cells of a plethora of organisms, ranging from plants, fishes, mice, to even humans. This has been conventionally achieved by using methodologies such as single nucleotide insertion/deletion in coding sequences, exon(s) deletion, mutations in the promoter region, introducing stop codon for protein truncation, and addition of foreign DNA for functional elucidation of genes. However, recent years have witnessed the advent of novel techniques that use programmable site-specific nucleases like CRISPR/Cas9, TALENs, ZFNs, Cre/loxP system, and gene trapping. These have revolutionized the field of experimental transgenesis as well as contributed to the existing knowledge base of classical genetics and gene mapping. Yet there are certain experimental/technological barriers that we have been unable to cross while creating genetically modified organisms. Firstly, while interfering with coding strands, we inadvertently change introns, antisense strands, and other non-coding elements of the gene and genome that play integral roles in the determination of cellular phenotype. These unintended modifications become critical because introns and other non-coding elements, although traditionally regarded as “junk DNA,” have been found to play a major regulatory role in genetic pathways of several crucial cellular processes, development, homeostasis, and diseases. Secondly, post-insertion of transgene, non-coding RNAs are generated by host organism against the inserted foreign DNA or from the inserted transgene/construct against the host genes. The potential contribution of these non-coding RNAs to the resulting phenotype has not been considered. We aim to draw attention to these inherent flaws in the transgenic technology being employed to generate mutant mice and other model organisms. By overlooking these aspects of the whole gene and genetic makeup, perhaps our current understanding of gene function remains incomplete. Thus, it becomes important that, while using genetic engineering techniques to generate a mutant organism for a particular gene, we should carefully consider all the possible elements that may play a potential role in the resulting phenotype. This perspective highlights the commonly used mouse strains and the most probable associated complexities that have not been considered previously, resulting in possible limitations in the currently utilized transgenic technology. This work also warrants the use of already established mouse lines in further research.

## Introduction

Traditionally, techniques involving the introduction of specific mutations/foreign DNA at the site of the targeted gene to either inactivate it or to correct a faulty gene have been one of the widely used approaches in modern biology utilized for functional elucidation of genes. Even today, these are routinely used as standard methods of choice to investigate vertebrate and invertebrate model organisms, such as mouse, plant, zebrafish, drosophila, nematode, and bacteria. In general, to study a gene function, the dominant-negative approach, knock-in, complete, partial, tissue-specific, and conditional knockout approaches are utilized based on the needs of the individual investigation. Moreover, recent advances in techniques involving CRISPR/Cas9 have not only expedited transgenesis but also rejuvenated the field of therapeutics as a potential tool in treating diseases like lung cancer as well as the ongoing pandemic, COVID-19 [5, 6, 36, 48]. Indeed, these techniques have proven to be powerful in understanding the minutiae of gene function, such as how a specifically located amino acid residue in a particular peptide and its corresponding DNA sequence in the gene play a crucial role in determining its function. For example, in knockin mouse model, p53 gene is engineered in a way that it harbors those mutations that are generally found in human sporadic cancer cases having either a mutant or a non-functional p53 gene [22]. Unsurprisingly, these mutations in humans cause different syndromes and cancers. Additionally, each respective mutation presents a distinct phenotype in mice, suggesting diversity in the mechanisms of p53 regulation in different microenvironments/tissues/genetic backgrounds. However, one cannot completely explain the difference in phenotypes produced by the same p53 mutation in both organisms based only on the difference in genes, species, and microenvironment.

Currently, we understand that central dogma alone cannot explain the behavior of the cell quite well, and complexity supersedes quantity. We now know that only a very small percentage (~ 2%) of our genome codes for functional proteins and that most of the genome still is beyond our limited understanding. The conventional view of the mammalian genome is that ~ 25,000 protein-coding genes are dispersed within a quite repetitive and largely non-transcribed sequence. Over the past decade, this view has been challenged by the discovery of several different and essential RNA species in mammalian cells that are termed as non-coding RNAs. This non-coding genome lies mixed and interspersed with the coding genome in such an intricate manner that today it is an extremely daunting task to discriminate between the two [51]. For instance, for functional proteins, coding regions tend to be much longer, and presence of an ORF (open reading frame) of at least 300 nucleotides (100 aa) is commonly used to define a transcript as “coding,” whereas many long transcripts with known non-coding functions may also typically contain multiple ORFs. These ORFs may give rise to proteins, might be translated inefficiently, or may even produce a non-functional protein which is rapidly degraded by proteasomes. These gray areas in defining coding and non-coding elements remain unexplored and may open new avenues of research. Even though we have begun to understand the signatures and properties of this tessellated non-coding entity, yet it is very early to anticipate or understand its full complexity.

## The problem

**Fig. 1:**
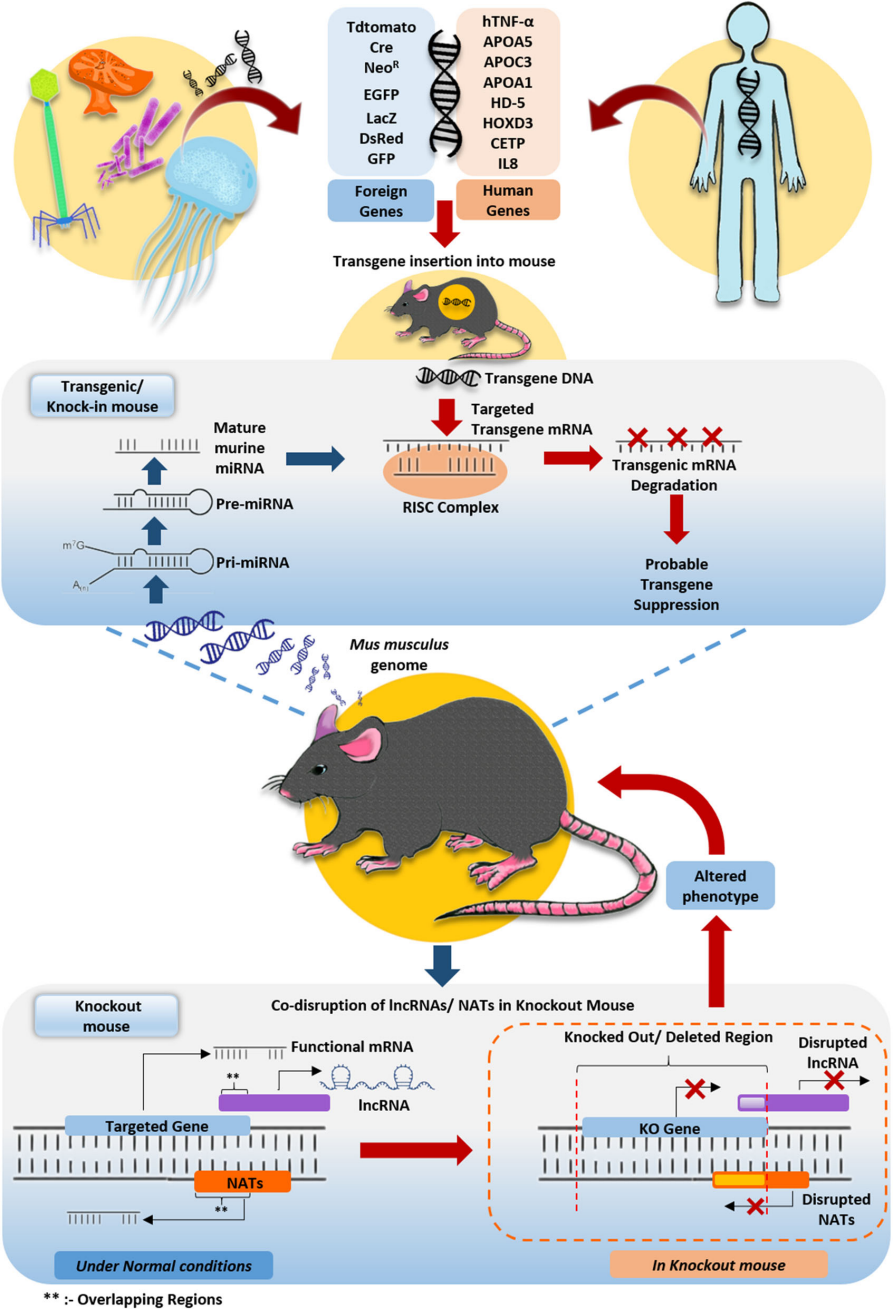
Probable mechanisms of inadvertent sequence changes in transgenic mice. Genes of foreign origin such as those from humans and marker genes become potential targets of murine miRNAs once expressed within the cells of knockin/transgenic mice. Non-coding elements such as NATs and lncRNAs may get co-disrupted along with the target gene and contribute to the resulting phenotype of the mice

The whole biology and engineering of “knocking out” genes become a little more complex per se due to the presence of important regulatory elements in the form of non-coding RNAs like miRNAs, lncRNAs, and natural antisense transcripts (NATs) inside and outside of the traditionally defined coding sequence (Fig. 1). Hence, it would be incorrect to state that knocking out a gene by the available traditional approaches will produce a phenotype that can precisely be attributed to the loss of that gene only. Until the end of last century and even currently, scientists have engineered numerous knockouts by deleting or modifying exon(s), e.g., by inserting reporter genes, by trapping the promoters and coding sequences, and by truncating the large part of protein by inserting a stop signal. However, the effect of unintentional alteration of several non-coding genes present within/outside the introns, and sometimes within exons, has not been taken into account in the process of knockout mouse generation. Moreover, the unintentional disruption of natural antisense transcripts (NATs) present in the non-coding strand of DNA during knockout generation further complicates the matter as they participate in various cellular regulatory processes via the *cis* or *trans* mechanisms, for instance, *Cftr* gene knockout mouse (*Cftr*^*−/−*^) which was generated by inserting an in-frame mutation in exon 10, to produce a truncated protein [47]. These *Cftr* knockout mice displayed a very strong phenotype, limiting their viability to a maximum of 40 days. The mouse *Cftr* gene has 28 exons, and there are several long intronic regions in the gene. Interestingly, a report published by Hill et al. on introns from *CFTR* demonstrated that introns alone are capable of coordinating the expression of functionally related genes [20]. They overexpressed three long intronic sequences (6a, 14b, and 23) from the *CFTR* gene in epithelial cells (HeLa), in which *CFTR* is not normally expressed. They observed that the expression of the *CFTR* introns caused extensive, specific, and highly reproducible transcriptional changes, affecting genes linked to *CFTR* function. Authors posited that, since these transfected cells do not express the *CFTR* protein-coding transcript, observed effects were certainly caused by the intronic sequences. Because all three intronic sequences do not include any known miRNAs or predicted stem-loop structures, they seem to act in *trans* as long ncRNA regulatory elements [20]. Similarly, constructs containing common selection markers/reporter genes like GFP, EGFP, Neo^r^, LacZ, and DsRed are often left within the target genome post-selection [9, 21, 29, 37, 62]. However, these genes themselves can become potential targets of miRNAs of host origin, e.g., *Mus musculus* as discussed later*.* Therefore, it would not be wrong to assume that the resulting phenotype can be attributed to the combined effect of “altering the specific coding gene” as well as the “other non-coding genes” that get affected inadvertently due to the disruption by genetic engineering method used to generate the knockout organism. This work attempts to highlight the presence and/or disruption of these non-coding elements.

## Analysis

Coding region or mRNA sequences of the transgenes were retrieved from the NCBI nucleotide database and used as target sequences for analysis. The custom miRNA prediction tool available at miRDB, an online database for miRNA target prediction [7], was utilized to search for *Mus musculus* miRNAs potentially targeting the mRNAs generated from commonly used reporter genes, Cre recombinase (Table 1), and human genes expressed in transgenic mouse models (Table 2). An arbitrary minimum cutoff value of 60 was selected for the target SCORE for selection of miRNAs in cases where several miRNAs with a wide range of scores were retrieved.

**Table 1:** *Mus musculus* miRNAs against inserted foreign genes and corresponding transgenic mice. Reporter genes like Neo^r^, LacZ, Cre recombinase, DsRed, and TdTomato have widely been used to generate transgenic mice. However, due to their non-mammalian origin, (e.g., DsRed from *Discosoma sp.*, GFP from *Aequorea victoria*, and LacZ from *Escherichia coli* K12), most of these genes may be potentially targeted by *Mus musculus* miRNAs once expressed in transgenic mice

Genes and accession numbers	Origin	miRNAs targeting the transcript	Example of mouse strains	References
TdTomato (LT009456)	*Discosoma sp.*	mmu-miR-3080-5p, mmu-miR-7019-3p, mmu-miR-1952, mmu-miR-3099-5p, mmu-miR-7659-5p, mmu-miR-1249-3p, mmu-miR-7008-5p, mmu-miR-6935-5p, mmu-miR-6922-5p, mmu-miR-7118-5p, mmu-miR-7115-5p, mmu-miR-5123, mmu-miR-6966-5p, mmu-miR-705, mmu-miR-6993-5p, mmu-miR-219b-5p, mmu-miR-6929-5p	Ai14(RCL-tdT)-D mice (stock No. 007914)	[56]
			Ai9(RCL-tdT) mice (stock No. 007909)	[27]
			mTmG mice (stock No. 007676)	[32]
Lac Z (b-D-galactosidase) (NC_000913)	*Escherichia coli*	mmu-miR-770-5p, mmu-miR-770-3p, mmu-miR-767, mmu-miR-1906, mmu-miR-203b-3p, mmu-miR-122-3p, mmu-miR-3071-3p, mmu-miR-7011-3p, mmu-miR-6372,mmu-miR-214-3p, mmu-miR-615-3p, mmu-miR-761	R26R mice (stock No. 003474)	[25]
			Z/EG transgenic mice (stock No. 004178)	[37]
			Z/EG transgenic mice (stock No. 003920)	[39]
Cre Recombinase (DQ023272)	*Escherichia virus P1*	mmu-miR-878-3p, mmu-miR-3084-3p, mmu-miR-221-5p, mmu-miR-6356, mmu-miR-7085-3p, mmu-miR-7243-5p, mmu-miR-219a-1-3p, mmu-miR-3070-5p, mmu-miR-3113-5p, mmu-miR-532-3p	Emx1^IRES *cre*^ mice (stock No. 005628)	[31]
			HSA-Cre79 mice (stock No. 006149)	[58]
			Adipoq-Cre mice (stock No. 010803)	[44]
			Agrp-Ires-cre mice (stock No. 012899)	[15]
			Albumin-Cre mice (stock No. 003574)	[54]
			Alb1-cre mice (stock No. 016832)	[30]
			Amh-cre transgenic mice (stock No. 007915)	[60]
			AQP2-Cre mice (stock No. 006881)	[59]
EGFP (U55761.1)	*Escherichia coli*	mmu-miR-7009-5p, mmu-miR-3098-3p, mmu-miR-509-5p, mmu-miR-7668-3p, mmu-miR-7036a-3p	Z/EG mice (stock No. 003920)	[63]
GFP (L29345)	*Aequorea victoria*	mmu-miR-804, mmu-miR-6979-3p, mmu-miR-5619-3p, mmu-miR-346-5p, mmu-miR-721, mmu-miR-6389,mmu-miR-6341, mmu-miR-301b-3p, mmu-miR-301a-3p, mmu-miR-130c, mmu-miR-130b-3p, mmu-miR-130a-3p, mmu-miR-344d-2-5p, mmu-miR-592-5p, mmu-miR-3094-3p, mmu-miR-9768-5p, mmu-miR-6967-3p, mmu-miR-3087-5p, mmu-miR-9-5p, mmu-miR-7093-3p, mmu-miR-7675-3p, mmu-miR-133a-5p, mmu-miR-669p-5p, mmu-miR-669 l-5p, mmu-miR-669f-5p, mmu-miR-669a-5p, mmu-miR-7683-3p, mmu-miR-7027-5p, mmu-miR-7657-5p, mmu-miR-486b-5p, mmu-miR-486a-5p, mmu-miR-6941-3p, mmu-miR-8092, mmu-miR-19b-3p, mmu-miR-19a-3p, mmu-miR-7663-3p, mmu-miR-494-5p, mmu-miR-410-5p	p25 Tg mice (stock No. 005706)	[29]
DsRed Express/MST/T1 (GQ268961)	*Discosoma sp.*	mmu-miR-7019-3p, mmu-miR-3080-5p, mmu-miR-1952, mmu-miR-7008-5p, mmu-miR-6935-5p, mmu-miR-6922-5p, mmu-miR-1249-3p, mmu-miR-219b-5p, mmu-miR-3099-5p, mmu-miR-7038-3p, mmu-miR-705, mmu-miR-6993-5p	DsRed.T3 transgenic mice (stock No. 005441)	[9]
			Dcx-DsRed transgenic mice (stock No. 009655)	[42]
			NG2DsRedBAC transgenic mice (stock No. 008241)	[18]
Neomycin Resistance Gene; Neo^r^ (NC_009980)	*Salmonella enterica*	mmu-miR-719, mmu-miR-323-5p	DR-4 transgenic mice (stock No. 003208)	[62]
			Z/EG transgenic mice (stock No. 003920)	[63]

**Table 2:** Human genes expressed in transgenic mice are targeted by murine miRNAs in corresponding transgenic mice. Human genes expressed in transgenic mice become potential targets of *Mus musculus* miRNAs due to their foreign nature. This miRNA-target mRNA interaction may often lead to interference with their expression in mice. The potentially targeting miRNAs were retrieved from miRDB using their custom target prediction tool

Genes and accession numbers	Function	miRNAs targeting the transcript	Example of mouse strains	References
hTNF-α (NM_000594)	Regulatory role in inflammatory response and host defense against bacterial infection.	mmu-miR-466 l-3p, mmu-miR-3967, mmu-miR-8093, mmu-miR-7224-3p, mmu-miR-504-3p, mmu-miR-5127, mmu-miR-150-5p, mmu-miR-7660-3p, mmu-miR-105, mmu-miR-7049-5p, mmu-miR-721, mmu-miR-6389, mmu-miR-6341, mmu-miR-301b-3p, mmu-miR-301a-3p, mmu-miR-130c, mmu-miR-130b-3p, mmu-miR-130a-3p, mmu-miR-7086-5p, mmu-miR-6418-5p, mmu-miR-210-5p, mmu-miR-6910-5p,	hTNFalpha transgenic mice	[26]
APOA5 (NM_052968)	Regulatory role in maintenance of plasma triglyceride levels.	mmu-miR-138-2-3p, mmu-miR-3098-3p, mmu-miR-3057-3p, mmu-miR-3085-3p, mmu-miR-3064-5p, mmu-miR-7081-3p, mmu-miR-6936-5p, mmu-miR-1894-5p, mmu-miR-7648-3p, mmu-miR-6940-5p, mmu-miR-6356, mmu-miR-361-3p, mmu-miR-762, mmu-miR-1964-5p, mmu-miR-7214-3p, mmu-miR-6979-5p	hAPOA5 transgenic mice	[16]
			hAPOAV transgenic mice	[38]
APOC3 (NM_000040)	Regulatory role in maintenance of plasma triglyceride levels. Inhibition of lipoprotein lipase and hepatic lipase.	mmu-miR-7042-3p, mmu-miR-7238-3p, mmu-miR-6899-3p, mmu-miR-6950-5p, mmu-miR-7216-5p, mmu-miR-6540-3p, mmu-miR-6979-5p, mmu-miR-1982-5p, mmu-miR-7672-5p, mmu-miR-127-5p, mmu-miR-3099-5p	human apoC-III Tg mice (stock No. 006907)	[61]
			hAPOC3 transgenic mice	[16]
APOA1 (NM_000039)	Involved in lipid/ cholesterol transport and metabolism.	mmu-miR-6392-5p, mmu-miR-5622-3p, mmu-miR-708-5p, mmu-miR-28a-5p, mmu-miR-5621-5p	Tg Hu ApoA1 mice (stock No. 001927)	[53]
HD-5 (NM_021010)	Cytotoxic/ antimicrobial protein involved in host defense against pathogens.	mmu-miR-3074-5p, mmu-miR-7240-5p, mmu-miR-3473a, mmu-miR-185-5p, mmu-miR-1933-3p, mmu-miR-882, mmu-miR-145a-3p, mmu-miR-203b-3p, mmu-miR-7217-3p, mmu-miR-1264-5p, mmu-miR-7229-3p, mmu-miR-881-5p, mmu-miR-691, mmu-miR-7216-5p, mmu-miR-539-5p, mmu-miR-1897-5p, mmu-miR-12186-5p, mmu-miR-665-3p, mmu-miR-6338, mmu-miR-29b-2-5p	HD-5 transgenic mice	[40]
HOXD3 (NM_006898)	Transcription factor that plays a regulatory role in morphogenesis.	mmu-miR-873a-5p, mmu-miR-7220-3p, mmu-miR-6370, mmu-miR-1968-5p, mmu-miR-495-3p, mmu-miR-12200-5p, mmu-miR-1192, mmu-miR-8118, mmu-miR-6394, mmu-miR-6367, mmu-miR-351-5p, mmu-miR-125b-5p, mmu-miR-125a-5p, mmu-miR-6387, mmu-miR-12186-3p, mmu-miR-1968-3p, mmu-miR-195b, mmu-miR-195a-5p, mmu-miR-7049-3p, mmu-miR-1948-5p, mmu-miR-1907, mmu-miR-6988-5p, mmu-miR-7226-5p, mmu-miR-6419, mmu-miR-6342, mmu-miR-497a-5p, mmu-miR-322-5p, mmu-miR-6353, mmu-miR-16-5p, mmu-miR-15b-5p, mmu-miR-15a-5p, mmu-miR-546, mmu-miR-497a-3p, mmu-miR-7016-5p, mmu-miR-3068-5p, mmu-miR-124b-3p	pHOXD3-transduced CD-1 mice	[8]
			p HA/HoxD3 transduced C57BL/KsJ-db/db mice	[19]
3’ UTR in CETP minigene (NM_000078)	Involved in cholesteryl ester and triglyceride transfer between lipids (HDL, VLDL, LDL).	mmu-miR-6396, mmu-miR-7016-5p, mmu-miR-6967-5p, mmu-miR-1943-5p, mmu-miR-149-3p, mmu-miR-770-3p, mmu-miR-6918-3p	CETP mice (stock No. 003904)	[41]
CDS (exon 1, 2, 13-16) in CETP minigene (NM_000078)		mmu-miR-6396, mmu-miR-487b-5p, mmu-miR-7016-5p, mmu-miR-6967-5p, mmu-miR-1943-5p, mmu-miR-149-3p, mmu-miR-3475-3p, mmu-miR-6972-5p, mmu-miR-449a-5p, mmu-miR-34a-5p		
IL 8/CXCL 8 (BC013615)	Chemokine involved in immune response by promoting chemotaxis and phagocytosis.	mmu-miR-140-3p, mmu-miR-6931-5p, mmu-miR-6951-5p, mmu-miR-7216-5p, mmu-miR-7675-3p, mmu-miR-497b, mmu-miR-3572-3p, mmu-miR-5101, mmu-miR-216c-5p, mmu-miR-3473f, mmu-miR-7683-3p, mmu-miR-7027-5p, mmu-miR-7116-3p, mmu-miR-682, mmu-miR-493-5p, mmu-miR-694, mmu-miR-7010-5p, mmu-miR-6951-3p, mmu-miR-376a-5p, mmu-miR-466i-3p, mmu-miR-7685-5p, mmu-miR-7670-5p, mmu-miR-212-5p, mmu-miR-466 l-3p, mmu-miR-669c-3p, mmu-miR-669d-3p, mmu-miR-467 g, mmu-miR-466e-3p, mmu-miR-466d-3p, mmu-miR-466a-3p, mmu-miR-297c-3p, mmu-miR-297b-3p, mmu-miR-297a-3p, mmu-miR-150-3p, mmu-miR-666-3p, mmu-miR-6964-5p, mmu-miR-691, mmu-miR-499-3p, mmu-miR-6998-3p, mmu-miR-7119-3p, mmu-miR-205-3p, mmu-miR-7221-3p, mmu-miR-6481, mmu-miR-6908-3p	IL-8Tg mice	[1]
hACE2 (NM_001371415)	Enzyme that catalyzes the hydrolysis of angiotensin II into angiotensin. Also serves as receptor for coronaviruses.	mmu-miR-6964-3p, mmu-miR-465d-5p, mmu-miR-465c-5p, mmu-miR-465b-5p, mmu-miR-465a-5p, mmu-miR-7054-5p, mmu-miR-6999-5p, mmu-miR-7665-3p, mmu-miR-26b-5p, mmu-miR-26a-5p, mmu-miR-1929-5p, mmu-miR-6978-3p, mmu-miR-6338, mmu-miR-29b-2-5p, mmu-miR-6951-5p, mmu-miR-7018-5p, mmu-miR-7680-5p, mmu-miR-7221-3p, mmu-miR-802-5p, mmu-miR-6914-3p, mmu-miR-1298-3p, mmu-miR-6994-5p	K18-hACE2 mice (stock No. 034860)	[57]
			hACE2 transgenic mice (ACTB promoter)	[49]
			Transgenic hACE2 mice (endogenous mouse Ace2 promoter)	[2]

A search of previously published literature was performed for knockout/mutant mice in which the introduction of specific mutations/foreign DNA at the site of the targeted gene had also inadvertently caused the disruption of lncRNAs or NATs. The affected genes and the co-disrupted non-coding elements were analyzed and complied with the publications which have utilized the mice (Table 3).

**Table 3:** Genes, NATs, lncRNAs, and corresponding mice. List of mouse strains showing probable inadvertent partial/complete disruption of overlapping sequence of NATs on the antisense strand along with intended target genes

Genes and gene ID	Function	NAT/lncRNA	Example of mouse strains	References
IL-1α (16175)	Regulatory role in immune response by promoting inflammation.	AS IL-1α	C57BL/6 J-Il1a^em1(Luc-eGFP)Smoc^ mice	[14]
			B6N(Cg)-*Il1a*^*tm1b(EUCOMM)Wtsi*^/J (stock No.027517)	[46]
HoxD-3 (15434)	Transcription factor that plays a regulatory role in morphogenesis	Hoxd3os1	Hoxd3^−/−^ mice	[12]
			Hoxa3^−/−^;hoxd3^−/−^ mice	[13]
Igf2r (16004)	Receptor for insulin-like growth factor 2 and mannose 6-phosphate. Involved in intracellular lysosomal enzyme trafficking.	Airn	Igf2r mutant mice	[33]
Dlx-1 (13390)	Homeobox transcription factor involved in craniofacial patterning.	Dlx-1as	*STOCK Dlx1/Dlx2*^*tm1.1Rth*^/J (stock No. 025612)	[45]
Msx1 (17701)	Transcriptional repressor that plays a major regulatory role during early embryogenesis.	Msx1as	Msx1^flox^ mice (stock No. 025204)	[17]

## Results

### Commonly used foreign genes targeted by *Mus musculus* miRNAs

Neomycin resistance gene (Neo^r^) is one of the widely utilized selection markers for the cells which are correctly targeted, and the neomycin cassette itself is normally left within the genome post-selection, assuming that it has no adverse effect on the eukaryotic cell biology [21, 50, 62]. But upon careful observation, it can be seen that the Neo^r^ gene construct itself is a potential target of several miRNAs of the eukaryotic origin or more specifically the miRNAs within the cells of the neomycin cassette containing transgenic mice (Table 1). Similarly, lacZ is another widely used reporter molecule, and its gene is often used in generating transgenic mice. A simple analysis revealed a similar fate of the lacZ gene as another strong target of several murine microRNAs (Table 1). Several other reporter genes that are widely used in mouse transgenic technology such as GFP, EGFP, TdTomato, and DsRed also have been shown as potential targets of murine microRNAs (Table 1). Hence, it can be correctly assumed that any gene that contains the Neo^r^/lacZ/GFP/EGFP/TdTomato/DsRed variants can also be considered as de novo targets of microRNAs of murine origin. Interestingly, one of the most widely used recombinase enzyme, Cre, which is used in mice studies for fate mapping, stem cell homing, and gene deletion, is also a potential target of several murine microRNAs (Table 1). Using the miRDB custom prediction tool [7], we searched for potential *Mus musculus* miRNAs that could target the abovementioned foreign genes that are frequently used in the generation of transgenic mice strains (Table 1). Based on the analyzed data, we propose that the resulting phenotype produced by interfering with the gene of interest may not solely be due to the disruption of that particular gene but due to the combined interference of the gene of interest and the associated non-coding elements. Additionally, these reporter genes or other elements of a targeting vector that are deliberately left in the mouse may very well act as sponges/sinks for the miRNAs or other non-coding RNAs, thus interfering with the normal physiology of the cell.

### Human genes in transgenic mice targeted by *Mus musculus* miRNAs

Over the last three decades, transgenic mice expressing human genes have proven to be an efficient tool to model human diseases. These murine models have successfully accelerated the drug discovery process as well as contributed to the knowledge base of the underlying molecular mechanisms of those diseases [23, 28]. However, due to the foreign nature of human genes being expressed in these mouse models, they often may become targets of murine miRNAs which may interfere with their expression in mice, for example, the *CETP* gene containing mouse or APOE*3-Leiden. CETP mouse is widely used in atherosclerosis research and has been very useful in understanding lipid metabolism and drug discovery [24, 52]. Mice naturally lack *cetp* gene, but these transgenic mice express the human *CETP* gene. Interestingly, our analysis indicates that 3’UTR, as well as the coding sequence of this gene, are potential targets of several murine microRNAs (e.g., mir149-3p) (Table 2). Similarly, in the three strains of transgenic mice expressing human *ACE2* gene currently being utilized to model the effect of SARS-CoV-2, the inserted *hACE2* gene CDS is also a potential target of murine miRNAs (Table 2) [34, 35, 49, 57]. Other mice expressing human *TNF-α*, *IL-8*, *APOA1*, *APOA5*, and *HD5* are some more examples, where the murine microRNAs are targeting the inserted human genes (Table 2). Using the miRDB custom prediction tool [7], we retrieved murine miRNAs that can potentially target the aforementioned human genes commonly expressed in transgenic mice to model human disease. Our data predicts that the observed phenotype in these mice may not explicitly be a result of only the inserted transgene, but rather a combined effect of the inserted transgene and the endogenous microRNAs acting on the foreign gene.

### Co-disruption of natural antisense transcripts (NATs) and long non-coding RNAs (lncRNAs) with the gene of interest in knockout mice

Recent years have seen a rising number of studies investigating the role of natural antisense transcripts (NATs) in eukaryotes. This has shed light on their *cis-* as well as *trans-*activity in gene regulation at various levels and NATs have been shown to play a crucial regulatory role in eukaryotic gene expression [3, 55, 64]. Generally, these are non-protein-coding fully processed mRNAs that are transcribed from the opposite strand of protein-coding sense transcripts [4]. In currently used transgenic techniques, while introducing mutations in the target site of the gene of our interest, we often not only disrupt the sequence of our target gene but also the partially/completely overlapping sequence of genes for NATs on the antisense strand. Although the disruption of NATs may be inadvertent, it interferes with its *cis-*/*trans-*activity. Hence, the resulting knockout phenotype would have to be attributed to the disruption of both the target gene and the corresponding overlapping NAT sequence. This should make us reconsider the assignment of the “bonafide mutant for the target gene only” status to the transgenic mice generated in such cases. We performed a literature search for such mice with co-disruption of target genes and overlapping NATs and found several such cases (Table 3). For instance, *Hoxd-3* knockout mice have been created by insertion of pD3Neo2TK vector carrying 11.7 kb of *Hoxd-3* sequence with disruption of *Hoxd-3* at nucleotide 82 of exon 1 by an MC1neo poly-A cassette [12]. Murine *Hoxd-3* has 3 exons and 2 introns and has a 5’ end overlap (4137 bp) with its antisense regulatory element “hoxd3os1” and the disruption of exon1 (size 324 bp) also results in the disruption of intron 2 in “hoxd3os1” due to the overlap. Hence, the resulting phenotype should be attributed to the disruption of both of these elements. Similarly, double-mutant mice were created with a targeted disruption in hoxa-3 and hoxd-3 in which the resulting phenotype would be due to the similar nature of disruption of hoxd-3 [13]. Another example of NATs disruption in genetically engineered mice is “Airn” in Igf2r mutant mouse. Igf2r has 48 exons and has a 28,395 bp overlap with its natural antisense transcript “Airn,” a long non-coding RNA. This mouse gene is responsible for silencing the insulin-like growth factor 2 receptor gene and flanking genes in the mice. The overlap spans exon 1, exon 2, intron 1, and a major portion of intron 2. Igf2r knockout mice were created by replacing 0.33 kb of 5’ flanking sequence and 38 codons of exon 1 by a neomycin resistance gene (Neo^r^) cassette [33]. This would also replace a portion of intron 1 of Airn and hence contribute to the phenotype originally attributed to the disruption of the only Igf2r. Similarly in Dlx-1/2 floxed conditional knockout mice, Dlx-1 has a 3343 bp overlap with its natural antisense transcript “Dlx-1as” spanning exons 2 and 3 and intron 2 completely and a portion of intron 1. These mice have been generated by introducing *loxP* sites located between exons 1 and 2 of both Dlx-1 and 2 genes (found in the opposite orientation on chromosome 2, 9427 bp apart from each other) [45]. Dlx-1/2 floxed mice were crossed with Olig1-Cre knockin mice which completely excised exons 2 and 3 and intron 2 of each gene and the intervening ~ 10 kbp sequence (which contains Dlx-1as on the complement strand in that region). Therefore, the deletion of entire Dlx-1as would also contribute to the resulting phenotype along with the deletion of Dlx-1 and 2. In Msx-1 conditional KO mice, Msx-1, a 4059 bp long homeobox gene, has a 2187 bp overlap with its natural antisense transcript “Msx1os” spanning portions of exon 2 and the single intron of Msx-1. Conditional KO mice of Msx-1 and 2 have been generated by introducing 2 *loxP* sites flanking exon 2 of each gene and crossing them with Msx-2Cre mice to obtain a global knockout of Msx-1 and 2 [17]. Although there was no apparent effect of the *loxP* sites present within intron and sequence downstream to exon 2 on Msx-1 and 2 gene functions, no account of disruption of “Msx1os” in the region opposite to intron (of the complementary strand) due to *loxP* site insertion has been reported. Post, global deletion of exon 2 via cre excision, the change in phenotype would have to be attributed to both disruption/knockout of Msx-1 as well as Msx1os. Several other mammalian gene-NAT pairs have been reported elsewhere [43]. These analyses demonstrate that the inadvertent disruption of NATs has been completely missed from the rigorous scheme of transgenesis and warrants a re-look into the biology that is affected by it.

## Conclusion

Technically, this is a limitation of the biological system itself that we may never be able to overcome. In most cases, man-made mutations introduced into the mouse genome would ultimately affect both strands of DNA and hence, the non-coding genes, whereas a natural mutation in the form of point mutation may not affect the other strand. However, when a natural mutation/deletion is affecting a large part of a chromosome, we must acknowledge the phenotype as a collective representation of both coding and non-coding gene disruptions. This can also be seen in mice where unknown modifiers from different genetic backgrounds interact with the same targeted gene to contribute to anomalous differences in the phenotype. For example, in the first documented case describing the influence of genetic background on gene expression, diabetes (db) and obese (ob) mutations against a B6 background were shown to only cause obesity and transient diabetes, but, on a C57BLKS/J (BKS) background, they caused obesity and severe diabetes [10, 11]. However, in addition to the modifier genes, we might also be seeing the effects of these non-coding genes, which play essential roles in cellular processes that get affected due to genetic deletions. Contrary to this, often we observe that knocking out a gene does not produce expected results. Commonly, this is explained as the gene not being crucial for either development or maintenance. However, one can argue that altering the coding gene at one locus gets compensated by the simultaneous loss of non-coding gene(s) at the same position. The African proverb “When elephants fight, it is the grass that suffers,” explains the fate of “non-coding genes” well. Because of our incomplete understanding of the complexity of non-coding entities in the past, there is a strong possibility that these components of the genome were inadvertently affected while engineering knockout mice. Hence, it becomes extremely critical to revisit the old methods of generating knockouts with our current understanding of the concepts and examine the transgenic strategy and affected gene functions more carefully. Nevertheless, the development of strategies to single out a particular gene function without affecting other associated non-coding elements will be a highly complex task. However, it should be noted that this may not be necessarily true for all the knockouts created to date. Our work warrants the use of already established mice lines in further research.

## Data Availability

NA.

## Abbreviations

TALENs: Transcription activator-like effector nucleases
CRISPR: Clustered regularly interspaced short palindromic repeats
Cas9: CRISPR-associated protein 9
ZFN: Zinc finger nuclease
Cre: (Causes Recombination) Recombinase Enzyme
loxP: Locus of crossing (x) over P1
CFTR: Cystic fibrosis transmembrane conductance regulator
CETP: Cholesteryl ester transfer protein
KO: Knockout
ACTB: Actin, Beta (*Gallus gallus*)
lncRNA: Long non-coding RNAs
miRNAs: Micro RNAs
GFP: Green florescent protein
EGFP: Enhanced green florescent protein
tdtomato: Tandem-dimer tomato
Neo^r^: Neomycin resistance gene
LacZ: β-galactosidase encoding gene lactose operon
Dsred: *Discosoma sp*. Red
hTNF-a: Human tumor necrosis factor alpha
APOA5: Apolipoprotein A-V
APOC3: Apolipoprotein C-III
APOA1: Apolipoprotein A-I
HD-5: Human alpha defensin 5
HOXD3: Homeobox D3
IL8: Interleukin 8

## References

[1] Asfaha S, Dubeykovskiy AN, Tomita H (2013). Mice that express human interleukin-8 have increased mobilization of immature myeloid cells, which exacerbates inflammation and accelerates colon carcinogenesis. Gastroenterology.

[2] Bao L, Deng W, Huang B, et al (2020) The pathogenicity of SARS-CoV-2 in hACE2 transgenic mice. Nature 583:830–833. 10.1038/s41586-020-2312-y 10.1038/s41586-020-2312-y 32380511

[3] Barrett LW, Fletcher S, Wilton SD (2012). Regulation of eukaryotic gene expression by the untranslated gene regions and other non-coding elements. Cell Mol Life Sci.

[4] Beiter T, Reich E, Williams RW, Simon P (2009). Antisense transcription: a critical look in both directions. Cell Mol Life Sci.

[5] Burgio G (2018). Redefining mouse transgenesis with CRISPR/Cas9 genome editing technology. Genome Biol.

[6] Chekani-Azar S, Gharib Mombeni E, Birhan M, Yousefi M (2020). CRISPR/Cas9 gene editing technology and its application to the coronavirus disease (COVID-19), a review. J Life Sci Biomed.

[7] Chen Y, Wang X (2020). miRDB: an online database for prediction of functional microRNA targets. Nucleic Acids Res.

[8] Chen Y, Xu B, Arderiu G (2004). Retroviral delivery of homeobox D3 gene induces cerebral angiogenesis in mice. J Cereb Blood Flow Metab.

[9] Choi SY, Bae H, Jeong S-H (2020). YAP/TAZ direct commitment and maturation of lymph node fibroblastic reticular cells. Nat Commun.

[10] Coleman DL (1978). Obese and diabetes: two mutant genes causing diabetes-obesity syndromes in mice. Diabetologia.

[11] Coleman DL, Hummel KP (1973). The influence of genetic background on the expression of the obese (Ob) gene in the mouse. Diabetologia.

[12] Condie BG, Capecchi MR (1993). Mice homozygous for a targeted disruption of Hoxd-3 (Hox-4.1) exhibit anterior transformations of the first and second cervical vertebrae, the atlas and the axis. Development.

[13] Condie BG, Capecchi MR (1994). Mice with targeted disruptions in the paralogous genes hoxa-3 and hoxd-3 reveal synergistic interactions. Nature.

[14] de Menezes MN, Salles ÉM, Vieira F (2019). IL-1α promotes liver inflammation and necrosis during blood-stage Plasmodium chabaudi malaria. Sci Rep.

[15] Engström Ruud L, Pereira MMA, de Solis AJ (2020). NPY mediates the rapid feeding and glucose metabolism regulatory functions of AgRP neurons. Nat Commun.

[16] Fruchart-Najib J, Baugé E, Niculescu L-S (2004). Mechanism of triglyceride lowering in mice expressing human apolipoprotein A5. Biochem Biophys Res Commun.

[17] Fu H, Ishii M, Gu Y, Maxson R (2007). Conditional alleles of Msx1 and Msx2. Genesis.

[18] Grubb S, Cai C, Hald BO (2020). Precapillary sphincters maintain perfusion in the cerebral cortex. Nat Commun.

[19] Hansen SL, Myers CA, Charboneau A (2003). HoxD3 accelerates wound healing in diabetic mice. Am J Pathol.

[20] Hill AE, Hong JS, Wen H (2006). Micro-RNA-like effects of complete intronic sequences. Front Biosci a J virtual Libr.

[21] Hussain S-RA, Yalvac ME, Khoo B, et al (2020) Adapting CRISPR/Cas9 System for Targeting Mitochondrial Genome. bioRxiv 2020.02.11.944819. 10.1101/2020.02.11.944819 10.3389/fgene.2021.627050 PMC8055930 33889176

[22] Iwakuma T, Lozano G (2007). Crippling p53 activities via knock-in mutations in mouse models. Oncogene.

[23] Jiang R-D, Liu M-Q, Chen Y (2020). Pathogenesis of SARS-CoV-2 in transgenic mice expressing human angiotensin-converting enzyme 2. Cell.

[24] Jiang XC, Agellon LB, Walsh A (1992). Dietary cholesterol increases transcription of the human cholesteryl ester transfer protein gene in transgenic mice. Dependence on natural flanking sequences. J Clin Invest.

[25] Kakiuchi N, Yoshida K, Uchino M (2020). Frequent mutations that converge on the NFKBIZ pathway in ulcerative colitis. Nature.

[26] Keffer J, Probert L, Cazlaris H (1991). Transgenic mice expressing human tumour necrosis factor: a predictive genetic model of arthritis. EMBO J.

[27] Kim J-E, Fei L, Yin W-C (2020). Single cell and genetic analyses reveal conserved populations and signaling mechanisms of gastrointestinal stromal niches. Nat Commun.

[28] King MR, Anderson NJ, Deciu M (2020). Insulin deficiency, but not resistance, exaggerates cognitive deficits in transgenic mice expressing human amyloid and tau proteins. Reversal by Exendin-4 treatment. J Neurosci Res.

[29] Klein H-U, McCabe C, Gjoneska E (2019). Epigenome-wide study uncovers large-scale changes in histone acetylation driven by tau pathology in aging and Alzheimer’s human brains. Nat Neurosci.

[30] Lau-Corona D, Bae WK, Hennighausen L, Waxman DJ (2020). Sex-biased genetic programs in liver metabolism and liver fibrosis are controlled by EZH1 and EZH2. Plos Genet.

[31] Laukoter S, Beattie R, Pauler FM (2020). Imprinted Cdkn1c genomic locus cell-autonomously promotes cell survival in cerebral cortex development. Nat Commun.

[32] Le V, He Y, Aldahl J (2020). Loss of androgen signaling in mesenchymal sonic hedgehog responsive cells diminishes prostate development, growth, and regeneration. Plos Genet.

[33] Ludwig T, Eggenschwiler J, Fisher P (1996). Mouse mutants lacking the type 2 IGF receptor (IGF2R) are rescued from perinatal lethality in Igf2 and Igf1r null backgrounds. Dev Biol Press.

[34] McCray PB, Pewe L, Wohlford-Lenane C (2007). Lethal infection of K18-hACE2 mice infected with severe acute respiratory syndrome coronavirus. J Virol.

[35] Muñoz-Fontela C, Dowling WE, Funnell SGP (2020). Animal models for COVID-19. Nature.

[36] Nair J, Nair A, Veerappan S, Sen D (2020). Translatable gene therapy for lung cancer using Crispr CAS9—an exploratory review. Cancer Gene Ther.

[37] Nayak G, Zhang KX, Vemaraju S (2020). Adaptive thermogenesis in mice is enhanced by opsin 3-dependent adipocyte light sensing. Cell Rep.

[38] Pennacchio LA, Olivier M, Hubacek JA (2001). An apolipoprotein influencing triglycerides in humans and mice revealed by comparative sequencing. Science.

[39] Sakamoto T, Obara N, Nishikii H (2019). Notch signaling in nestin-expressing cells in the bone marrow maintains erythropoiesis via macrophage integrity. Stem Cells.

[40] Salzman NH, Ghosh D, Huttner KM (2003). Protection against enteric salmonellosis in transgenic mice expressing a human intestinal defensin. Nature.

[41] Schuster S, Rubil S, Endres M (2019). Anti-PCSK9 antibodies inhibit pro-atherogenic mechanisms in APOE*3Leiden.CETP mice. Sci Rep.

[42] Shen M, Wang F, Li M (2019). Reduced mitochondrial fusion and Huntingtin levels contribute to impaired dendritic maturation and behavioral deficits in Fmr1-mutant mice. Nat Neurosci.

[43] Shendure J, Church GM (2002). Computational discovery of sense-antisense transcription in the human and mouse genomes. Genome Biol.

[44] Siang DTC, Lim YC, Kyaw AMM (2020). The RNA-binding protein HuR is a negative regulator in adipogenesis. Nat Commun.

[45] Silbereis JC, Nobuta H, Tsai H-H (2014). Olig1 function is required to repress dlx1/2 and interneuron production in Mammalian brain. Neuron.

[46] Skarnes WC, Rosen B, West AP (2011). A conditional knockout resource for the genome-wide study of mouse gene function. Nature.

[47] Snouwaert JN, Brigman KK, Latour AM (1992). An animal model for cystic fibrosis made by gene targeting. Science.

[48] Syding LA, Nickl P, Kasparek P, Sedlacek R (2020). CRISPR/Cas9 epigenome editing potential for rare imprinting diseases: A Review. Cells.

[49] Tseng C-TK, Huang C, Newman P (2007). Severe acute respiratory syndrome coronavirus infection of mice transgenic for the human angiotensin-converting enzyme 2 virus receptor. J Virol.

[50] Tucker KL, Wang Y, Dausman J, Jaenisch R (1997). A transgenic mouse strain expressing four drug-selectable marker genes. Nucleic Acids Res.

[51] Ulitsky I, Bartel DP (2013). lincRNAs: genomics, evolution, and mechanisms. Cell.

[52] Van den Maagdenberg AM, Hofker MH, Krimpenfort PJ (1993). Transgenic mice carrying the apolipoprotein E3-Leiden gene exhibit hyperlipoproteinemia. J Biol Chem.

[53] Viaud M, Abdel-Wahab O, Gall J (2020). ABCA1 exerts tumor-suppressor function in myeloproliferative neoplasms. Cell Rep.

[54] Wang G, Xu J, Zhao J (2020). Arf1-mediated lipid metabolism sustains cancer cells and its ablation induces anti-tumor immune responses in mice. Nat Commun.

[55] Wight M, Werner A (2013). The functions of natural antisense transcripts. Essays Biochem.

[56] Wilson DH, Jarman EJ, Mellin RP (2020). Non-canonical Wnt signalling regulates scarring in biliary disease via the planar cell polarity receptors. Nat Commun.

[57] Winkler ES, Bailey AL, Kafai NM (2020). SARS-CoV-2 infection of human ACE2-transgenic mice causes severe lung inflammation and impaired function. Nat Immunol.

[58] Xiong L, Zhao K, Cao Y (2020). Linking skeletal muscle aging with osteoporosis by lamin A/C deficiency. Plos Biol.

[59] Xu C, Wang F, Chen Y (2020). ELABELA antagonizes intrarenal renin-angiotensin system to lower blood pressure and protects against renal injury. Am J Physiol Physiol.

[60] Zhang H, Chen F, Dong H (2020). Loss of Fbxw7 in Sertoli cells impairs testis development and causes infertility in mice†. Biol Reprod.

[61] Zhang Y, He W, He C (2019). Large triglyceride-rich lipoproteins in hypertriglyceridemia are associated with the severity of acute pancreatitis in experimental mice. Cell Death Dis.

[62] Zhou H, Zeng Z, Koentgen F (2019). The testicular soma of Tsc22d3 knockout mice supports spermatogenesis and germline transmission from spermatogonial stem cell lines upon transplantation. Genesis.

[63] Zhu Y, Hirata T, Mackay F, Murakami F (2020). Chemokine receptor CXCR7 non-cell-autonomously controls pontine neuronal migration and nucleus formation. Sci Rep.

[64] Zinad HS, Natasya I, Werner A (2017). Natural antisense transcripts at the interface between host genome and mobile genetic elements. Front Microbiol.

